# 

**Published:** 1867-04

**Authors:** 


### LIST OF PAYMENTS.

Drs. W. F. Benson, $4; Surg. General, $7; D. L. Pores, $4; J. W. Crawford, $4; J. M. Williams, $4; J. P. Taylor, $3; Frank H. Hamelton, $4; R. P. Tally, $4; J. C. Turnipseed, $4; S. V. D. Hill, $3.50; H. H. Minor, $3.50; A, Reynolds, $4; J. A. Johnson, $4; A. H. Jones, $4; N. B. Drewry, $4; W. S. R. Hardman, $4; J. M. McLaughlin, $1; W. H. Johnson, $4; D. W. Hammond, $4; J. E. Wendell, $1; W. T. Goldsmith, $4; W. H. Pope, $4; A. H. McCants, $2; John Coston, $2; J. M. Boring, $4; J. W. Bailey, $4; J. E. Bacon, $4; J. C. Avery, $4; Wm. O Daniel, $4.50; Carlton, $4; Moore, $4; Long, $4; J. A. Vanzant, $4; G W. MoDowell, $4; Milton Bishop, $4; J. J. Kent, $4; R. A. Bethune, $4; J, R. Ray, $4; Robert Harper, $4; Jno. Dickinson, $4; F. M. Thomason, $4; J. H. Spier, $2; J. S. Branch, $4; W. H. Chatham, $2; Cromwell & Connally, $4; J. Guild, $4; J. E. Walker, $4; C. R. Lamply, $4; J. C. Turnipseed, $2; Morrison, $4; W. Willingham, $4; J. S. Wilson, $2; Hays & Franklin, $4; J. C. Phelphs, $4; G. W. Adamson, $2; L. L. Lincicum, $2.

